# Oral squamous cell carcinoma: a clinicopathological study on 194 cases in northeastern Brazil. A cross-sectional retrospective study

**DOI:** 10.1590/1516-3180.2017.0293061217

**Published:** 2018-03-16

**Authors:** Amanda Almeida Leite, Augusto César Leal da Silva Leonel, Jurema Freire Lisboa de Castro, Elaine Judite de Amorim Carvalho, Pablo Agustin Vargas, Luiz Paulo Kowalski, Danyel Elias da Cruz Perez

**Affiliations:** I MSc. Student, Oral Pathology Unit, Piracicaba Dental School, Universidade Estadual de Campinas (UNICAMP), Piracicaba (SP), Brazil.; II MSc. Student, Oral Pathology Unit, School of Dentistry, Universidade Federal de Pernambuco, Recife (PE), Brazil.; III PhD. Professor, Oral Pathology Unit, School of Dentistry, Universidade Federal de Pernambuco (UFPE), Recife (PE), Brazil.; IV PhD. Professor, Oral Pathology Unit, School of Dentistry, Universidade Federal de Pernambuco (UFPE), Recife (PE), Brazil.; V PhD. Professor, Oral Pathology Unit, Piracicaba Dental School, Universidade Estadual de Campinas (UNICAMP), Piracicaba (SP), Brazil.; VI PhD. Director, Department of Otorhinolaryngology and Head and Neck Surgery, A.C. Camargo Cancer Center, Sao Paulo (SP), Brazil.; VII PhD. Professor, Oral Pathology Unit, School of Dentistry, Universidade Federal de Pernambuco (UFPE), Recife (PE), Brazil.

**Keywords:** Brazil, Mouth, Mouth neoplasms, Carcinoma, squamous cell

## Abstract

**BACKGROUND:**

Only a few studies have evaluated the clinicopathological features of oral squamous cell carcinoma (SCC) in Brazil, and most were conducted in the most industrialized region of the country, i.e. the southeastern region. The aim of this study was to evaluate the clinicopathological features of this malignant neoplasm in northeastern Brazil.

**DESIGN AND SETTING:**

Retrospective study performed in an oral pathology laboratory in Recife, Brazil.

**METHODS:**

All cases of oral SCC that occurred between 2000 and 2015 were studied. Clinical data were recorded and histological slides were reviewed. Statistical analysis was performed using the chi-square test (P ≤ 0.05).

**RESULTS:**

A total of 194 cases were evaluated. The male-to-female ratio was 1.5:1. The mean age was 65.4 years, and only 6.6% of the cases occurred in patients younger than 41 years. Most tumors consisted of well-differentiated SCC (54.6%).

**CONCLUSIONS:**

The findings of this study highlight the higher prevalence of oral SCC among women and the increasing number of cases among young patients. Thus there is no specific risk group for oral SCC, as in the past. This fact needs to be taken into consideration in clinical routine care, so that apparently innocuous malignant lesions do not go unnoticed in these individuals.

## INTRODUCTION

Squamous cell carcinoma (SCC) is a well-recognized malignant neoplasm that is responsible for more than 90% of oral malignancies.^1^^,^^2^^,^^3^^,^^4^ Worldwide, 405,000 new cases of oral cancer are expected every year.^3^ It is the seventh most common type of cancer among Brazilians.^5^

The etiology of these tumors is multifactorial. Tobacco and alcohol consumption are the most determinant etiological factors, especially when acting synergistically.^3^^,^^5^ Other associated factors include excessive exposure to ultraviolet light (for lip carcinomas), diets lacking fruits and vegetables, poor oral hygiene and betel nut chewing (especially in Asian populations). Although the true prevalence remains a matter of debate, high-risk HPV infection has also been described as an etiological factor.^5^^,^^6^

Although the epidemiological profile has changed over time, with considerable regional variations,^6^ there is still higher prevalence of oral cancer among males, which is consequential to greater exposure to risk factors among men. Oral SCC usually affects older individuals, after the fifth decade of life,^4^ with a reported mean age of 62 years.^2^^,^^5^ The usual clinical presentation is a painless ulcer on the border of the tongue or floor of the mouth.^2^^,^^5^

Only a few studies have evaluated the clinicopathological features of oral SCC in Brazil, and most of these were conducted in the most industrialized region of the country, i.e. the southeastern region.^7^^,^^8^^,^^9^ Thus, the objective of this study was to evaluate the clinicopathological features of oral SCC diagnosed in a less economically developed geographic area using the records from an oral pathology laboratory in the northeastern region of Brazil.

## METHODS

This study was approved by the local research ethics committee, under protocol no. 44536715.8.0000.5208. This was a cross-sectional, retrospective study.

All cases of squamous cell carcinoma that were diagnosed in the Oral Pathology Laboratory of the Federal University of Pernambuco, Recife, Brazil, between 2000 and 2015, were included in the study. All clinical records were reassessed, and the main clinical and epidemiological parameters were collected, including gender, age, place of origin of the patient, patient’s occupation, symptoms reported by the patient at the initial consultation, duration of complaints, tumor location, clinical presentation of the lesion, tumor size, first diagnostic hypothesis and presence of deleterious habits (smoking, alcohol consumption, or both).

In the same way as done in previous studies,^1^^,^^10^ the variable of age was subdivided into six groups: patients aged less than 41 years, between 41 and 50, between 51 and 60, between 61 and 70, between 71 and 80 and over 80. The locations of the tumors included the following regions: border of oral tongue, alveolar mucosa and gingiva (including the retromolar area and buccal mucous fold), floor of mouth and ventral tongue, hard/soft palate, lower lip (including mucosa and vermilion) and buccal mucosa.

The clinical aspects of the lesions were classified into four groups: ulcerated, leukoplakia, leukoerythroplakia and lesions that presented an ulcerated component with areas of leukoerythroplakia. The symptoms were divided into two groups: the first group comprised patients who presented any painful symptoms relating to the lesion (including spontaneous or induced pain); and the second group comprised patients who were asymptomatic at the initial consultation.

All cases were reviewed under a microscope. The histopathological analysis and grading were performed in accordance with the classification proposed by the World Health Organization (WHO) in 2017,^11^ based on the degree of cell differentiation. The tumors were classified as follows: well-differentiated, when they showed tissue architecture similar to the normal pattern of the squamous epithelium; moderately differentiated, when they presented some degree of nuclear pleomorphism and mitotic activity, and little keratinization; and poorly differentiated, when they presented predominance of immature cells, numerous typical and atypical mitoses, and minimal keratinization. All microscopic SCC variants were also considered in the study.

The data collected were analyzed using the Statistical Package for the Social Sciences (SPSS), version 20.0. Descriptive statistical analysis was performed for all variables described above. Cases for which data were not available in the clinical records, or were incomplete, were not included in the analysis. The variables were analyzed by means of the chi-square test, taking the significance level to be 5% (P < 0.05). For the statistical analysis, the numerical variables were grouped, as follows: age (≤ 45 years and > 45 years); duration of complaints (< 3 months, 3-6 months and > 6 months); and tumor size (2.0 cm, 2.1-4.0 cm and > 4.0 cm).

## RESULTS

Out of the 4,727 oral lesions diagnosed over a 15-year period, 250 were malignant (5.28%). From these, 194 cases (77.6%) were squamous cell carcinomas. The majority of the cases evaluated (97.4%) came from the public health service, and the remaining from private services (2.6%).

Most cases occurred in men, with 118 cases (60.8%), representing a male-to-female ratio 1.5:1. The age group most affected was the seventh and eighth decades of life (51.9%). The mean age among the cases was 65.4 years and the age range was from 26 to 94 years. Twelve cases (6.6%) were in patients under 41 years of age.

The border of the tongue was the most common site (51 cases; 26.7%), followed by the floor of the mouth/ventral tongue (36 cases; 18.8%) and the alveolar mucosa/gingiva/retromolar area (32 cases; 16.8%). The hard/soft palate and the lower lip were affected in 25 (13.1%) and 21 cases (11%), respectively.

Most patients (112; 68.7%) reported a duration of complaints of up to 6 months. Complaints for periods over 12 months had the lowest prevalence, with 22 cases (13.5%). Clinically, ulcerated lesions (76 cases; 46.3%) were most commonly observed, followed by leukoplakia (30 cases; 18.3%) and leukoerythroplakia (20 cases; 12.2%). Most lesions (76 cases; 76.0%) were of sizes smaller than 4 cm; among these, 38 (38%) were lesions smaller than 2.0 cm and the remaining 38 (38%) were lesions between 2.1 and 4.0 cm. Lesions over 4.0 cm were present in 24 cases (24.0%) (Table 1).

**Table 1:** Clinical features, occupation and risk factors among patients with oral squamous cell carcinomas in the sample studied

Variables	Number of cases	%
Gender (n = 194)		
Male	118	60.8
Female	76	39.2
Age (n = 181)		
< 41 years	12	6.6
41 to 50 years	20	11.0
51 to 60 years	40	22.1
61 to 70 years	47	25.9
71 to 80 years	47	25.9
> 80 years	15	8.3
Tumor site (n = 191)		
Border of oral tongue	51	26.7
Floor of mouth/ventral tongue	36	18.8
Alveolar mucosa/gingiva/retromolar area	32	16.8
Hard/soft palate	25	13.1
Lower lip	21	11
Buccal mucosa	14	7.3
Others	12	6.3
Duration of complaints (n = 163)		
< 3 months	65	39.9
4-6 months	47	28.8
7 to 12 months	29	17.8
> 12 months	22	13.5
Clinical appearance (n = 164)		
Ulcer	76	46.3
Leukoplakia	30	18.3
Leukoerythroplakia	20	12.2
Ulcer with leukoerythroplakia areas	17	10.4
Other	22	12.9
Tumor size (n = 100)		
< 2.1 cm	38	38.0
2.1 to 4.0 cm	38	38.0
4.1 to 6.0 cm	17	17.0
> 6.0 cm	7	7.0
Occupation (n = 130)		
Retired	37	28.5
Farmer	30	23.1
Housewife	15	11.5
Mason	10	7.7
Housekeeper	7	5.4
Others	31	23.8
Patients exposed to risk factors (n = 58)*		
Tobacco	28	48.3
Alcohol	1	1.7
Tobacco and alcohol	29	50.0

*Only in 58 clinical records was the patient’s exposure to the main risk factors (tobacco and alcohol) reported.

The diagnostic hypothesis most frequently reported by the practitioners was squamous cell carcinoma, in 145 cases (78.0%). Leukoplakia (5 cases; 2.7%) was the second most cited hypothesis, followed by actinic cheilitis (3 cases; 1.6%). Other hypotheses that were raised included erythroplakia, papilloma, traumatic ulcer, fibrous hyperplasia, lichen planus and frictional keratosis, which together represented 16.1% of the sample studied.

Regarding the patient’s occupation, most patients were retired (28.5%), followed by farmers (23.1%) and housewives (11.5%) (Table 1). Only in 58 clinical records was it reported that the patients were either exposed or non-exposed to the main risk factors (tobacco and alcohol). Among these, there were 28 cases of smokers, one of a chronic alcoholic patient, and 29 of exposure to both factors. Most (70.1%) of the clinical records, corresponding to 136 cases, did not include this information.

Patients who reported either spontaneous or induced pain accounted for 75 cases (38.7%). Thirty-five patients (12.9%) were asymptomatic. In the largest proportion of the cases (94; 48.5%), this information was not available.

Analysis on the histological grade showed that most cases consisted of well-differentiated SCC, representing 106 cases (54.6%). Moderately and poorly differentiated SCC represented, respectively, 72 cases (37.1%) and 10 cases (5.2%). Among the microscopic variants of SCC, two cases were diagnosed as verrucous carcinomas, two cases consisted of microinvasive carcinoma, one case consisted of basaloid SCC and one case was diagnosed as acantholytic SCC.

Correlations between the variables revealed that moderately differentiated SCC occurred more frequently on the border of the tongue and floor of the mouth (P < 0.05). Most cases that appeared in the form of leukoplakia were diagnosed in patients with durations of complaints of up to 6 months (P = 0.001). The number of smokers was significantly lower among the patients under 45 years of age (P = 0.02). The associations between age and occupation (P = 0.06), symptoms (P = 0.9), duration of complaints (P = 0.3), clinical features (P = 0.8), size of the tumor (P = 0.85) and histopathological grading (P = 0.45) were not significant. The associations between tumor size and gender (P = 0.58), occupation (P = 0.41), clinical features of the lesion (P = 0.06), duration of complaints (P = 0.34) and histopathological grading (P = 0.47) were not statistically significant. In addition, no association between duration of complaints and clinical features (P = 0.1) was observed, or between clinical features and histopathological grading (P = 0.3).

## DISCUSSION

In this study, the male-to-female ratio was 1.5:1, similar to what has been observed in other reports.^1^^,^^10^ Although higher prevalence among males is still commonly seen, it has been reported that this gender ratio is decreasing. This has been attributed to changes in the social context of women’s lives, such that they may be exposing themselves more significantly to the usual risk factors for oral SCC.^1^^,^^8^^,^^12^^,^^13^

SCC is usually a disease of elderly people, with peak incidence in the sixth and seventh decades of life.^1^^,^^7^^,^^12^ However, approximately 0.4 to 3.6% of oral cancers occur in young patients, i.e. those less than 40 years old.^14^ The incidence rises to close to 6% among patients up to 45 years old.^5^^,^^7^^,^^13^ Ribeiro et al.^7^ showed that the rate was 12% among patients in this age group. In the present study, the youngest patient was 26 years old, and 8.8% of the patients were under 45 years of age. Recent research has shown that the incidence of oral cancer in this younger population has been increasing significantly. The evidence suggests that the traditional risk factors would be less active in these patients, which thus draws attention to the influence of nutritional and biological factors such as HPV infection (especially HPV16). However, the mechanisms of viral action in these cases have not yet been well established.^7^^,^^8^^,^^13^

The increased risk of oral cancer among alcohol and tobacco users is well known.^9^^,^^12^ Consumption of both alcohol and tobacco together has a multiplicative effect and this has been implicated in about 75% of all head and neck SCC cases.^12^ In the present study, 50% of all the patients who were asked whether they used alcohol or tobacco said that they used both of these substances. Among the patients who were asked this question, 48.3% were smokers. However, this information was not reported by the majority (70.1%) of the practitioners who sent the specimens for histopathological diagnosis, thus showing the professionals’ negligence regarding obtaining this information. These data emphasize that there is a need to guide practitioners regarding the importance of constantly providing educational guidance on oral cancer to patients. Such guidance is currently the main way of preventing the disease. Nevertheless, despite the missing information, the confirmed data indicated that the percentage of smokers and drinkers was lower than in previous studies,^15^^,^^16^ in which the proportion of smokers and drinkers among the patients was up to 90%. Recent trends have shown results similar to those of the present investigation.^17^

The most frequent clinical characteristic in the initial consultation was the presence of an ulcer (46.3%), and this was similar to the findings of Pires et al.^8^ However, SCC may appears as white or red lesions, which are characterized as leukoplakia, erythroplakia or leukoerythroplakia.^13^ Although many cases of SCC do not show previous evidence of a potentially malignant disorder,^13^ it is essential that practitioners, especially dentists, should be aware of this group of lesions. In the present study, 18.3% of the cases consisted of leukoplakia and the majority of the patients had complained about the lesion for a maximum time of 6 months. Pain may account for 30 to 40% of complaints among patients with oral SCC, which usually is related to lesions at an advanced clinical stage.^2^

The clinical presentation of oral SCC can vary considerably and the initial tumors can often be subtle and asymptomatic, which may represent a diagnostic challenge in this early clinical stage.^13^ Nevertheless, more recent studies have shown that patients with oral cancer have been diagnosed at an earlier stage of disease.^8^^,^^12^ Those findings were similar to what was observed in the present study, in which 76% of the cases presented tumor sizes smaller than 4 cm at the initial consultation. Considering that those studies were also conducted at specialized diagnostic and treatment services, it needs to be considered that this scenario of findings was influenced by the health surveillance provided by these centers to these populations. On the other hand, it should also be considered that the information filled out by the professional, in some cases, might not represent the real size of the lesion. In the present series, in 60% of the cases, the duration of complaints reported by the patients was more than six months, thus probably indicating larger tumors.

It has been reported that patients delay their complaints about issues of this nature for approximately six months, although this length of time is variable.^18^ It has been estimated that such delays, for more than three months, lead to significant worsening of the prognosis.^19^ Affective factors such as fear and denial, and cultural issues, may be associated with delay in seeking a healthcare professional.^20^ Moreover, it was found in another study that most patients believed that the lesion would not be serious and that it would be resolved without any treatment.^21^ In addition to the patient’s delay, there may be a professional delay of between 1 and 5 months. Although there was no information on professional delays in the present study, it has been shown that limitation of the oral examination to teeth and gums and lack of knowledge of oral mucosal lesions may constitute factors that are associated with delays in diagnosing oral SCC.^18^

Oral SCCs are usually histologically graded as well or moderately differentiated,^1^^,^^8^^,^^10^ as observed in the present series. The histopathological grading usually shows weak influence on the prognosis for the lesions, mainly because of the subjective nature of the evaluation and the small size of biopsies in tumors that have great heterogeneity.^22^ In the present study, moderately differentiated cases were significantly more frequently diagnosed on the border of the tongue and the floor of the mouth, and such cases are known to have more dubious prognoses.^23^ There was no correlation between histopathological grading and the age of the patients affected.

Although this series presented interesting and relevant data, it is important to highlight some limitations of the study. Its retrospective design and consequent scope for missing information in relation to risk factors like smoking and alcohol, in addition to absence of data on neck status, treatment and outcome, prevented a more complete analysis. Despite this, the study revealed important clinicopathological and demographic features of an oral SCC series in a Brazilian region that had been poorly studied. This data may be valuable for practitioners in planning preventive measures and diagnosing the disease.

## CONCLUSION

The findings of this study highlight the higher prevalence of oral SCC among women and the increasing numbers of cases among young patients. Thus, the current trends indicate that there is no specific risk group for oral SCC, as in the past. This fact needs to be taken into consideration in clinical routine care, so that apparently innocuous malignant lesions do not go unnoticed in these individuals. Moreover, these findings are similar to observed in other Brazilian regions.
